# Bio-Priming with *Bacillus* Isolates Suppresses Seed Infection and Improves the Germination of Garden Peas in the Presence of *Fusarium* Strains

**DOI:** 10.3390/jof10050358

**Published:** 2024-05-17

**Authors:** Dragana Miljaković, Jelena Marinković, Gordana Tamindžić, Dragana Milošević, Maja Ignjatov, Vasiljka Karačić, Snežana Jakšić

**Affiliations:** 1 Institute of Field and Vegetable Crops, 21000 Novi Sad, Serbia; jelena.marinkovic@ifvcns.ns.ac.rs (J.M.); gordana.tamindzic@ifvcns.ns.ac.rs (G.T.); dragana.milosevic@ifvcns.ns.ac.rs (D.M.); maja.ignjatov@ifvcns.ns.ac.rs (M.I.); snezana.jaksic@ifvcns.ns.ac.rs (S.J.); 2 Faculty of Agriculture, University of Belgrade, 11080 Belgrade, Serbia; vasiljka.dragic@gmail.com

**Keywords:** *Bacillus subtilis*, *Bacillus amyloliquefaciens*, *Fusarium* biocontrol, *Pisum sativum* L., seed bio-priming

## Abstract

Seed infection caused by *Fusarium* spp. is one of the major threats to the seed quality and yield of agricultural crops, including garden peas. The use of *Bacillus* spp. with multiple antagonistic and plant growth-promoting (PGP) abilities represents a potential disease control strategy. This study was performed to evaluate the biocontrol potential of new *Bacillus* spp. rhizosphere isolates against two *Fusarium* strains affecting garden peas. Six *Bacillus* isolates identified by 16S rDNA sequencing as *B. velezensis* (B42), *B. subtilis* (B43), *B. mojavensis* (B44, B46), *B. amyloliquefaciens* (B50), and *B. halotolerans* (B66) showed the highest in vitro inhibition of *F. proliferatum* PS1 and *F. equiseti* PS18 growth (over 40%). The selected *Bacillus* isolates possessed biosynthetic genes for endoglucanase (B42, B43, B50), surfactin (B43, B44, B46), fengycin (B44, B46), bacillomycin D (B42, B50), and iturin (B42), and were able to produce indole-3-acetic acid (IAA), siderophores, and cellulase. Two isolates, *B. subtilis* B43 and *B. amyloliquefaciens* B50, had the highest effect on final germination, shoot length, root length, shoot dry weight, root dry weight, and seedling vigor index of garden peas as compared to the control. Their individual or combined application reduced seed infection and increased seed germination in the presence of *F. proliferatum* PS1 and *F. equiseti* PS18, both after seed inoculation and seed bio-priming. The most promising results were obtained in the cases of the bacterial consortium, seed bio-priming, and the more pathogenic strain PS18. The novel *Bacillus* isolates may be potential biocontrol agents intended for the management of *Fusarium* seed-borne diseases.

## 1. Introduction

The rising demand of a growing world population for food, accompanied by the impact of climate change, puts increasing pressure on agroecosystems to improve production and sustainability [1]. The garden pea (*Pisum sativum* L.) has multiple functions in this context. Besides serving as a global source of food, pea plants fix atmospheric nitrogen, and thus their inclusion in cropping systems reduces the overuse of chemical fertilizers and improves soil properties [2,3]. It is a widely cultivated vegetable crop known for its great nutritional and health benefits due to its high-quality proteins, carbohydrates, vitamins, minerals, and various bioactive compounds [4]. Moreover, peas have gained momentum in organic production because of their nitrogen fixation ability, market potential, and profitability [5]. The annual global production of garden peas is approximately 20 million tons, with an area harvested of 2.5 million hectares and a yield of 7.8 t ha^−1^ [6]. The main garden pea producers in the world are China and India, followed by Pakistan, France, and USA [6]. In order to increase the productivity of garden peas, it is necessary to improve seed quality through all stages of production [7].

Healthy seeds are the main prerequisites for crop production and food security since numerous pathogens that reduce yield quantity and quality appear on and in seeds [8]. Problems in seed health could occur during production, storage, and transport, while the most serious infections and major economic losses associated with legumes have been reported for fungal pathogens. Among the various diseases affecting peas, wilts and rots caused by the fungi of the genus *Fusarium* represent one of the major factors limiting production worldwide. They pose a direct or indirect threat to seeds by causing seed rot and discoloration, while seed germination may be partially or even entirely hindered. Furthermore, *Fusarium* spp. can cause serious damage at all stages of crop growth and total yield losses in highly infested fields. Recently, *Fusarium* species were detected on garden pea seeds, with up to 10% of infected seeds per sample during routine quality control [9]. Since peas are mostly used as food, an additional concern is seed contamination with fungal biomass and the production of *Fusarium* mycotoxins [10].

Control measures, such as deep plowing, rotation, and soil sterilization are not efficient enough in reducing the occurrence and severity of *Fusarium* diseases. Furthermore, the use of synthetic fungicides is still the most effective control method with active substances such as fludioxonil, fludioxonil and metalaxyl-M, carbendazim, difenoconazole, and thiram. However, chemical control gradually leads to resistance and a race shift in *Fusarium* populations. Therefore, a series of strategies based on seed treatments with biocontrol agents (BCAs) have been developed to prevent the application of synthetic pesticides and to reduce the detrimental effects of agrochemicals on the environment and human health [11]. Biological control is a widely available, environmentally friendly, and inexpensive method for the suppression of plant pathogenic microorganisms, and is of special importance in organic vegetable production [12]. *Bacillus* spp. with antimicrobial activities are most commonly employed as BCAs to increase crop resilience against biotic stresses [12,13]. These bacteria are highly advantageous in achieving desired biocontrol effects, along with stimulating the growth and development of the host plant. *Bacillus* spp. inhibit the growth of fungal pathogens through various mechanisms, including the production of antimicrobial compounds, extracellular hydrolytic enzymes, competition, and inducing systemic resistance [13]. Moreover, *Bacillus* spp. play important roles in plant growth promotion and stress tolerance [14]. 

Seed treatments with *Bacillus* spp. may suppress seed infection and protect seedlings from pathogen intrusion during emergence and the initial growth stages, contributing to healthy crops and high yields. Bio-priming integrates bacterial inoculation with seed hydration by soaking seeds in bacterial suspension for a specific amount of time, which enhances bacterial colonization, activates quicker imbibition, and triggers the metabolic processes related to germination [15]. Thus, bio-priming provides better control against plant pathogens as compared to conventional treatments and increases seed germination, seedling vigor, and plant tolerance to biotic stressors [16]. Moreover, different seed priming techniques, including bio-priming, are often used to improve germination and vigor under abiotic stresses [17]. Bio-priming uses beneficial bacteria to promote seed and plant attributes through the production of regulatory substances, enhancement of nutrient uptake, and the protection of seedlings from soil- and seed-borne pathogens [18]. Several studies have reported the positive effects of different seed priming treatments on garden peas [19,20,21]. The application of *Bacillus*-based BCAs through bio-priming has recently emerged as a promising approach to controlling numerous plant diseases. However, there is a lack of information about the use of *Bacillus* biocontrol strains as bio-priming treatments in suppressing garden pea seed infection caused by seed-borne *Fusarium* strains.

The aim of this study was to determine the antifungal activity of 46 new *Bacillus* spp. isolates from different rhizosphere soils against four seed-borne *Fusarium* spp. strains affecting garden pea (*F. proliferatum* PS1, *F. equiseti* PS18), common bean (*F. proliferatum* P1), and soybean (*F. graminearum* S1). In addition, the antagonistic properties of *Bacillus* isolates were determined through the PCR detection of biosynthetic genes for lipopeptide antibiotics and hydrolytic enzymes. Moreover, the plant growth-promoting (PGP) properties and the effect of selected *Bacillus* spp. isolates on seed germination and the initial growth of garden peas were evaluated. Finally, the potential of the best-performing *Bacillus* spp. isolates through seed inoculation and seed bio-priming to improve germination in the presence of fungal infection (*F. proliferatum* PS1 and *P. equiseti* PS18) and reduce pathogen incidence on garden pea seeds was examined.

## 2. Materials and Methods

### 2.1. Bacillus Isolates

*Bacillus* spp. were isolated from rhizosphere soil collected from different locations and different hosts in Serbia. Samples included the rhizosphere of several field and vegetable crops cultivated on soils with different physical, chemical, and biological properties, tillage, and cropping backgrounds. Rhizosphere samples were sampled together with plant roots and transferred to the laboratory in sterile bags for the subsequent isolation of bacteria. Briefly, soil suspensions (1 g of sample in 9 mL of 0.9% *w*/*v* NaCl) were diluted (10^−2^–10^−7^), spread on nutrient agar (NA), and incubated at 28 °C for 24–48 h. Single bacterial colonies were sub-cultured and characterized according to morphological and biochemical properties [22]. All bacterial isolates were rod-shaped, catalase-, and Gram-positive. A total of 46 newly isolated strains showed certain antagonism towards different fungal pathogens, including *Fusarium* spp., and were selected for this study. Bacterial cultures were maintained on NA slants at 4 °C.

### 2.2. Pathogenic Fungi

Pathogenic fungi were obtained from the Laboratory for Seed Testing of the Institute of Field and Vegetable Crops (IFVCNS), Serbia. Four strains of *Fusarium* were tested: two strains, *F. proliferatum* PS1 and *F. equiseti* PS18, were isolated from symptomatic garden pea seeds [9], and the other two strains, *F. proliferatum* P1 and *F. graminearum* S1, were isolated from the infected common bean and soybean seeds, respectively [23,24]. The pathogens were isolated during routine seed health analyses, while seeds originated from the Rimski Šančevi experimental field (45°20′00″ N, 19°51′00″ E) in Serbia. Briefly, 400 seeds per sample were surface sterilized (1% NaOCl for 5 min), plated onto potato dextrose agar (PDA), and incubated at 23 ± 2 °C for 7 days. Fungal colonies were sub-cultured, morphologically identified, and tested for their pathogenicity [25]. PCR analysis of the translation elongation factor 1-α (*tef-1 α*) sequence with the primer pair EF1 (5′-ATGGGTAAGGACAAGAC-3′) and EF2 (5′-GGAAGTACCAGTGATCATGTT-3′) confirmed that the selected pathogenic strains belong to the abovementioned *Fusarium* species. Fungal cultures were maintained on PDA slants at 4 °C.

### 2.3. Antifungal Assay

The antifungal activity of *Bacillus* spp. isolates against four *Fusarium* strains (*F. proliferatum* PS1 and P1, *F. equiseti* PS18, and *F. graminearum* S1) were tested in vitro using a dual culture assay with slight modifications [26]. Bacterial isolates were cultivated in nutrient broth (NB) at 30 °C overnight, while fungal strains were grown on PDA at 23 ± 2 °C for 7 days. A bacterial culture was streaked through the center of the PDA plate, while the mycelial disc of the fungal strain (6 mm) was transferred on both sides of the Petri dish at equal distances from the bacterium (3 cm). The plates without bacterial inoculation were used as a control. Dual cultures and controls (three repetitions per treatment) were incubated at 25 °C for 7 days. The percent of growth inhibition (PGI) was calculated based on the following formula:

$$PGI\left(\%\right)=\frac{C-T}{C}\times 100$$

where C and T represent mycelium diameter (mm) in control and dual culture, respectively.

### 2.4. Molecular Identification of Bacillus Isolates

*Bacillus* spp. isolates were selected for molecular identification according to the results of an antifungal assay. DNA was extracted using a DNeasy Mini Kit (QIAGEN Inc., Hilden, Germany) from bacterial cultures grown on NA plates at 30 °C for 24 h. The polymerase chain reaction (PCR) was performed in 50 μL aliquots (25 μL of DreamTaq Green PCR MasterMix (Thermo Scientific, Waltham, MA, USA), 1 μL of forward and 1 μL of reverse primer, 2 μL of DNA template and 21 µL of nuclease-free water (Invitrogen, Waltham, MA, USA)) in thermal cycler (Eppendorf, Hamburg, Germany). Amplification of the 16S rDNA gene fragments was performed by using 27F (5′-AGAGTTTGATCMTGGCTCAG-3′) and 1492R (5′-TACGGYTACCTTGTTACGACTT-3′) primers. The PCR conditions were as follows: denaturation (94 °C, 5 min); 30 cycles of denaturation (94 °C, 30 s), annealing (50 °C, 1 min), extension (72 °C, 30 s), and final extension (72 °C, 7 min) [27]. Separating the PCR products was conducted by electrophoresis in agarose gel (1.5%) with ethidium bromide staining for DNA visualization, while purification and sequencing were performed in Macrogen Europe (Amsterdam, The Netherlands). The obtained DNA sequences of selected *Bacillus* isolates were analyzed using FinchTV Version 1.4. and the Nucleotide BLAST Tool (http://www.ncbi.nlm.nih.gov/BLAST (accessed 12 January 2024) at the National Center for Biotechnology Information (NCBI). Sequences of selected *Bacillus* spp. isolates and related *Bacillus* strains (GenBank, NCBI) were subjected to phylogenetic analysis using the neighbor-joining (NJ) method within MEGA 7 [28].

### 2.5. Detection of Antagonistic Traits

The characterization of *Bacillus* spp. isolates for antifungal traits included the determination of hydrolytic enzymes and lipopeptide antibiotics. The detection of biosynthetic genes encoding the production of hydrolytic enzymes, i.e., chitinase (*ChiA*) and endoglucanase, as well as lipopeptide antibiotics, i.e., surfactin (*Sfp*), iturin (*ItuA-ItuB*), bacillomycin D (*BamC*), and fengycin (*FenD*), was performed through PCR analysis. The primers were specific to *ChiA*, *Sfp*, *BamC*, and *FenD* target genes, viz. Qchi-f/Qchi-r, Sfp-f/Sfp-r, Bacc1-f/Bacc1-r, and FenD1-f/FenD1-r (Supplementary Materials Table S1), were previously described by Aydi Ben Abdallah et al. [29]. The primers specific for the endoglucanase gene, i.e., EN1F/EN1R (Supplementary Materials Table S1), were reported by Ashe et al. [30]. The PCR mixture (25 μL) consisted of 12.5 μL of DreamTaq Green PCR MasterMix, 0.5 μL of forward and reverse primer, 2 μL of DNA template, a concentration of 0.1–1 μg, and 9.5 µL of nuclease-free water. The PCR parameters for the detection of the *ChiA* gene were as follows: denaturation (94 °C, 4 min); 35 cycles of denaturation (92 °C, 1 min), annealing (58 °C, 1 min), extension (72 °C, 1 min) and final extension (72 °C, 7 min) [29]. Cycling parameters for PCR detection of *Sfp*, *BamC*, and *FenD* genes were as follows: denaturation (95 °C, 5 min); 30 cycles of denaturation (94 °C, 1 min), annealing (55 °C, 1 min), extension (72 °C, 1 min) and final extension (72 °C, 10 min) [29]. The part of the gene of the iturin operon (between *ItuA* and *ItuB*) was amplified using ItuP1-F/ItuP1-R primers (Supplementary Materials Table S1) described by Dimkić et al. [27], and the PCR conditions were as follows: initial denaturation (94 °C, 2 min); 10 cycles of auto-extension (94 °C, 15 s), primer annealing (45 °C, 15 s), and extension (68 °C, 3 min); 25 cycles of denaturation (94 °C, 15 s), primer annealing (45 °C, 15 s), and extension (68 °C, 3 min) and final extension (72 °C, 10 min). The cycling conditions for detection of the endoglucanase gene were as follows: initial denaturation (94 °C, 5 min); 10 cycles of touch down (94 °C, 30 s; 70 °C, 20 s; 74 °C, 45 s); 25 cycles of denaturation (94 °C, 30 s), annealing (60 °C, 20 s), extension (74 °C, 45 s) and final extension (74 °C, 10 min) [30]. The PCR products were electrophoresed as described earlier. Cellulase activity was assessed qualitatively by spot-inoculation of bacterial cultures on agar plates amended with 1% carboxymethyl cellulose (HiMedia, Mumbai, India) [31]. After incubation, cellulolytic activity was visualized by the appearance of clear zones around the inoculation spot.

### 2.6. Detection of Plant Growth-Promoting Traits

The ability of selected *Bacillus* isolates to produce indole-3-acetic acid (IAA) was tested by inoculating 1 mL of overnight-grown bacterial culture in NB enriched with L-tryptophan (250 and 500 µg mL^−1^) (HiMedia, Mumbai, India). After incubation at 30 °C for 24 h, the supernatant was mixed with Salkowski reagent (FeCl_3_-HClO_4_) in a ratio of 1:2 (*v*/*v*). The production of IAA was indicated by the color development after 20 min at room temperature and measured spectrophotometrically (UV/VIS Cary 60 E, Agilent, CA, USA) at 530 nm [32]. The phosphate (P) solubilization and P mineralization of the antagonistic isolates were detected on culture media supplemented with tricalcium phosphate (HiMedia, Mumbai, India) and lecithin (Thermo Fisher Scientific, Waltham, MA, USA), respectively [32]. The overnight-grown bacterial cultures were streaked on respective media, incubated, and evaluated for the development of halo zones around the colonies. Siderophore production was assessed on chromeazurol S (CAS) (HiMedia, Mumbai, India) [32]. The bacterial cultures were streaked on NA close to the edge with CAS agar, and their ability to produce siderophores was observed through changes in the color zones from blue to orange after 5 days of incubation at 28 °C. All tests were repeated three times, and in the case of positive results, the diameters of the zones were measured in mm.

### 2.7. Germination Assays

#### 2.7.1. Effect of Selected *Bacillus* Isolates on Germination and the Initial Growth of Non-Infected Garden Pea Seeds

The plant growth-promoting ability of selected *Bacillus* spp. isolates were examined in the first germination assay under controlled laboratory conditions. The seeds of the pea cultivar Dunav were obtained from the Department of Vegetable and Alternative Crops, IFVCNS, Serbia. Seeds were surface sterilized with 2% (*w*/*v*) sodium hypochlorite (Sigma Aldrich, St. Louis, MO, USA) for 5 min and rinsed with sterile distilled water. Inoculation was performed by soaking 100 seeds in a 10 mL bacterial suspension [32]. *Bacillus* isolates were cultivated in NB and incubated at 28 °C and 120 rpm (Edmund Bühler SM-30 B, Bodelshausen, Germany) for 24 h. Each bacterial suspension was adjusted to have a final concentration of 10^9^ colony-forming units per mL (CFU/mL). Each treatment was replicated three times (3 × 100 seeds). There were six bacterial treatments with the following *Bacillus* isolates: B42, B43, B44, B46, B50, and B66, along with a non-inoculated control. Treated seeds were placed in plastic boxes (240 × 150 mm) with moistened sterile sand and germinated under optimal conditions for garden peas at 20 °C with a 16/8 h day/night regime (Biobase BJPX-C300L, Jinan, China) [33]. The percentage of final germination (%), counting only seedlings with a well-developed root and shoot system, was determined 8 days after sowing. Ten normal seedlings from each replication were randomly selected to assess the growth-related parameters, including shoot and root length and dry weight, while the seedling vigor index (SVI) was calculated using the following formula:

SVI=SL×FG

where SL and FG represent seedling length (cm) and final germination (%), respectively.

#### 2.7.2. Effect of Selected *Bacillus* Isolates on Seed Germination and *Fusarium* Incidence on Infected Garden Pea Seeds

The second germination experiment was conducted in order to evaluate the ability of the best-performing *Bacillus* spp. isolates to improve the germination of infected garden pea seeds, along with their potential to reduce seed infection, i.e., pathogen incidence. This experiment was performed in vitro using the same pea cultivar used previously, two of the most effective *Bacillus* spp. isolates, and the *F. proliferatum* PS1 and *F. equiseti* PS18 strains. There were three bacterial treatments, i.e., B43, B50, and a consortium of the mentioned isolates (B43 + B50), each subsequently infected with a fungal pathogen. Two application techniques of bacterial antagonists, *viz.*, seed inoculation and seed bio-priming, were examined. Seed inoculation was performed by applying 1.5 mL of bacterial suspension (10^9^ mL^−1^) to the 30 seeds [34]. Seed bio-priming was conducted by immersing seeds in bacterial suspensions (1:5 *w*/*v*) for 5 h at 25 °C under dark conditions [35]. *Bacillus* isolates were cultivated in NB and incubated at 28 °C and 120 rpm (Edmund Bühler SM-30 B, Bodelshausen, Germany) for 24 h. Each bacterial isolate was grown individually and then mixed in equal proportions to form a consortium. The final concentration of bacterial suspensions was adjusted to 10^9^ CFU/mL. In both techniques, seed infection was performed by adding 1.5 mL of fungal suspension (10^5^ mL^−1^) to the *Bacillus*-treated seeds [34]. Seeds treated only with fungal suspension were designed as a positive control(s). The treated seeds were sown in Petri dishes (85 mm × 15 mm) onto filter paper soaked with sterile distilled water and incubated at 22 °C for one week in a light chamber (Biobase BJPX-C300L, Jinan, China) programmed for 16/8 h. Each treatment had four replications. The percentage of final germination (FG) was calculated according to the following formula:

$$FG\left(\%\right)=\frac{GS}{TS}\times 100$$

where GS and TS represent the number of germinated and total seeds, respectively [34].

The incidence (I) of *Fusarium* strains on garden pea seeds was estimated according to the following formula:

$$I\left(\%\right)=\frac{FS}{TS}\times 100$$

where FS and TS represent the number of seeds in which species occurred and the total number of seeds, respectively [36]. The incidence of *Fusarium* species on seeds was further classified as low (0–20%), moderate (21–50%), and high (over 50%) [36].

### 2.8. Statistical Analysis

The data were subjected to analysis of variance (ANOVA) followed by post hoc Tukey’s test to examine the differences between each treatment (*p* ≤ 0.05). The data were statistically processed by using STATISTICA 10.0 software (StatSoft Inc., Tulsa, OK, USA).

## 3. Results

### 3.1. Antifungal Activity of Bacillus spp. Isolates

**Figure 1:**
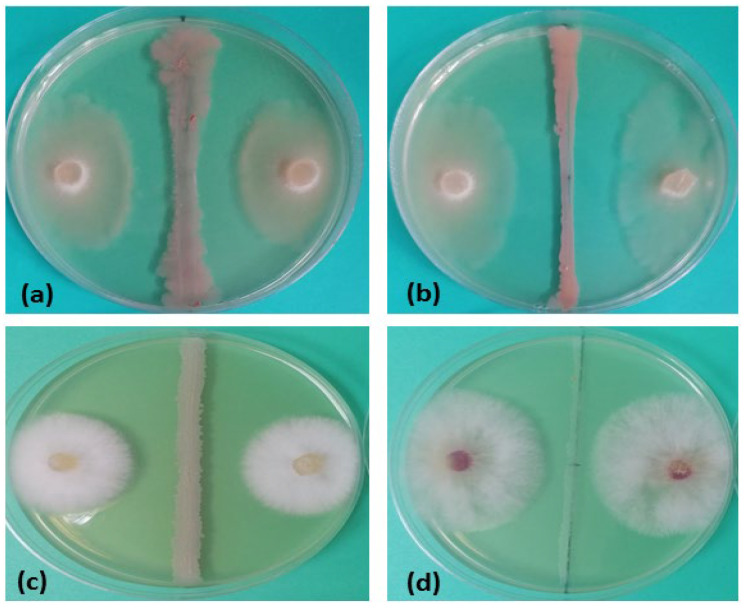
Antifungal effect of selected *Bacillus* spp. isolates. (**a**) B42 vs. *F. proliferatum* PS1, (**b**) B50 vs. *F. proliferatum* P1, (**c**) B43 vs. *F. equiseti* PS18, (**d**) B46 vs. *F. graminearum* S1.

A total of 46 indigenous *Bacillus* spp. isolates (B33–B78) from different rhizosphere soil samples were characterized according to in vitro antifungal activity, and the obtained results are presented in Table 1. The inhibition percentage of the fungal pathogen *F. proliferatum* PS1 ranged from 7.78% to 52.2%, with 39 active *Bacillus* isolates (Table 1, Figure 1a). The highest fungal growth inhibition was recorded against the *F. proliferatum* P1 strain, with 44 antagonistic *Bacillus* isolates and an inhibition percentage from 23.3% to 55.6% (Table 1, Figure 1b). Additionally, the inhibition percentage against *F. equiseti* PS18 and *F. graminearum* S1 ranged from 2.22% to 45.6% and 3.33% to 48.9%, with 36 and 38 antagonistic bacterial isolates, respectively (Table 1 and Figure 1c,d). Six *Bacillus* isolates, namely B42, B43, B44, B46, B50, and B66, with the highest average growth reduction in the four examined pathogenic strains (PGI ≥ 40%) were selected for molecular identification, further antifungal and plant growth-promoting characterization, and assays of garden pea seed germination.

### 3.2. Molecular Identification of Selected Bacillus Isolates

The selected isolates were identified by PCR amplification of the 16S rDNA gene after detection of the desired amplicon (1460 bp) and sequencing of the examined samples (Supplementary Materials Figure S1). The obtained nucleotide sequences were analyzed using Nucleotide BLAST and compared with the *Bacillus* ID database (NCBI). Six *Bacillus* isolates were identified as *B. velezensis* (B42), *B. subtilis* (B43), *B. mojavensis* (B44, B46), *B. amyloliquefaciens* (B50), and *B. halotolerans* (B66). The sequences with values 100% similar to those deposited in GenBank were submitted to the NCBI database (Table 2).

The phylogenetic analysis involved 17 nucleotide sequences of the isolated (6) and related (11) *Bacillus* species (Supplementary Materials Figure S2). A neighbor-joining tree shows that isolate B42 was grouped with *B. velezensis* XJ-C (MG752876.1) and M6 (MK226560.1) strains from the database. *Bacillus* isolate B43 was closely related to *B. subtilis* WJ-3 (KY368671.1) and ACHB-2 (KU867636.1) strains. Also, high homology was recorded between isolates B44 and B46 and strains *B. mojavensis* SWFU14 (KJ756333.1), A40 (OL636361.1), and N410 (MK629812.1); isolate B50 and strains *B. amyloliquefaciens* BA17 (MH891764.1) and BA31 (MG548650.1); isolate B66 and strains *B. halotolerans* ATCC 25096 (OQ876681.1) and XT-2 (MZ540321.1) (Supplementary Materials Figure S2).

### 3.3. Antagonistic Traits of Selected Bacillus Isolates

The results of in vitro cellulolytic activity and PCR detection of chitinase, endoglucanase, and lipopeptide genes are presented in Table 3. The ability of *Bacillus* spp. to produce extracellular cellulase was observed in 100% of the tested isolates. Five isolates, namely *B. velezensis* B42, *B. subtilis* B43, *B. mojavensis* B44 and B46, and *B. amyloliquefaciens* B50, were superior cellulase producers with halo zones larger than 10 mm (Table 3). PCR amplifications revealed the presence of genes encoding endoglucanase (1311 bp), surfactin (*Sfp*, 675 bp), bacillomycin D (*BamC*, 875 bp), iturin (*ItuA-ItuB*, 2000 bp), and fengycin (*FenD*, 964 bp) biosynthesis in 50%, 50%, 33.3%, 16.7%, and 33.3% of selected *Bacillus* isolates, respectively (Table 3, Supplementary Material Figures S3–S10). The chitinase gene (*ChiA*) was not detected in the examined *Bacillus* spp. (Table 3). The simultaneous presence of all four tested genes was not observed. Two of the biosynthetic genes were detected in isolates *B. velezensis* B42 (*BamC*, *ItuA-ItuB*), *B. mojavensis* B44 and B46 (*Sfp*, *FenD*), while one gene was observed in isolates *B. subtilis* B43 (*Sfp*) and *B. amyloliquefaciens* B50 (*BamC*). However, the tested lipopeptide genes were not found in isolate B66 (Table 3).

### 3.4. Plant Growth-Promoting (PGP) Traits of Selected Bacillus Isolates

The results of PGP characterization are presented in Table 4. Selected bacterial isolates were able to produce IAA in a range of 1.87–14.73 µg mL^−1^ and 2.80–15.70 µg mL^−1^ in medium with 250 and 500 µg mL^−1^ L-tryptophan, respectively. Isolates *B. subtilis* B43 and *B. amyloliquefaciens* B50 were the best IAA producers in both media. Selected *Bacillus* isolates were also positive for P-mineralization and siderophore production. On the other hand, the halo zones around the inoculation spot were not observed in the P-solubilization test.

### 3.5. Effect of Selected Bacillus Isolates on the Germination and Initial Growth of Non-Infected Garden Pea Seeds

The germination assay showed a significant effect of seed bacterization on the examined pea parameters (Table 5). Four isolates, namely *B. velezensis* B42, *B. subtilis* B43, *B. mojavensis* B44, and *B. amyloliquefaciens* B50, had a positive impact on final germination, leading to an increase from 1.33% to 4.33% as compared to control. The same four isolates, as well as isolate *B. mojavensis* B46, promoted the shoot length of treated pea seeds by 1.7–30.4% in relation to the non-treated seeds. All *Bacillus* isolates, except *B. velezensis* B42, had a stimulating effect on the root length of inoculated seeds, ranging from 0.5% to 23.5% compared to non-inoculated seeds. Furthermore, all bacterial treatments resulted in increases in shoot dry weight (2.3–19.5%) and root dry weight (3.3–15.4%). Seed inoculation with all *Bacillus* isolates except *B. mojavensis* B44 caused an improvement in seedling vigor index, ranging from 1.49% to 25.1%. A significant effect of particular isolates in relation to the control was recorded in the cases of shoot length (B50), root length (B43, B46, B50, B66), shoot dry weight (B43, B50), root dry weight (B50), and seedling vigor index (B43, B46, B50). Generally, isolates *B. subtilis* B43 and *B. amyloliquefaciens* B50 had a greater effect compared to other tested isolates, so their effect on garden pea seeds was further examined in the presence of the pea pathogens (*F. proliferatum* PS1 and *F. equiseti* PS18). Bacterial inoculants were further tested as single inoculants (B43, B50) and consortium (B43 + B50), assuming that their synergistic effects could contribute to higher survival, competitiveness, tolerance, and efficacy.

### 3.6. Effect of Selected Bacillus Isolates on the Germination of Infected Garden Pea Seeds

**Figure 2:**
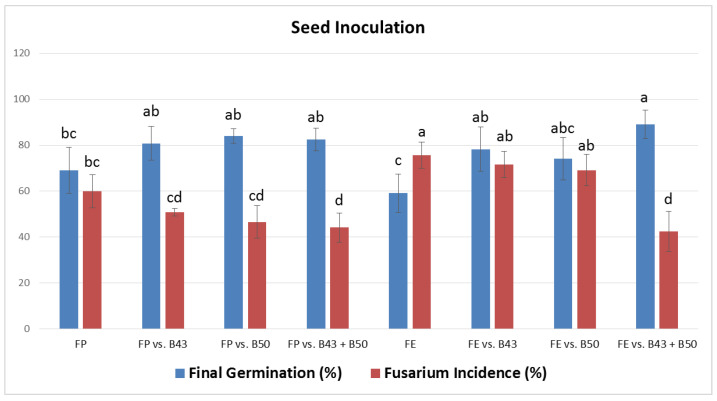
Final germination and pathogen incidence on garden pea seeds after inoculation with selected *Bacillus* isolates (*B. subtilis* B43, *B. amyloliquefaciens* B50 and B43 + B50 consortium) and infection with *F. proliferatum* PS1 (FP) and *F. equiseti* PS18 (FE). Means (*n* = 4) with standard deviation are shown. The values sharing the same lowercase letter within the same color are not significantly different (*p* ≤ 0.05, Tukey’s HSD test).

**Figure 3:**
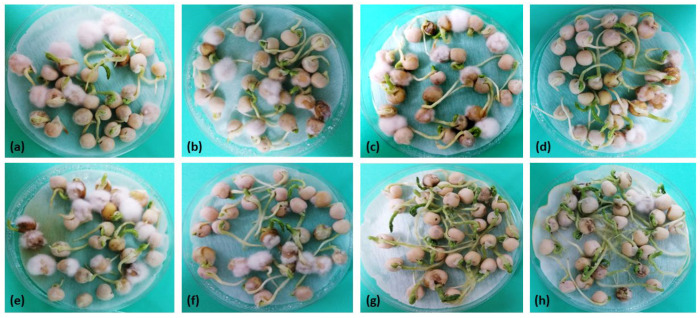
Antifungal effect of selected *Bacillus* isolates after seed inoculation. (**a**) *F. proliferatum* PS1- treated seeds, (**b**) *B. subtilis* B43 vs. PS1-treated seeds (**c**) *B. amyloliquefaciens* B50 vs. PS1-treated seeds, (**d**) B43 + B50 vs. PS1-treated seeds, (**e**) *F. equiseti* PS18-treated seeds, (**f**) *B. subtilis* B43 vs. PS18-treated seeds, (**g**) *B. amyloliquefaciens* B50 vs. PS18-treated seeds, (**h**) B43 + B50 vs. PS18-treated seeds.

**Figure 4:**
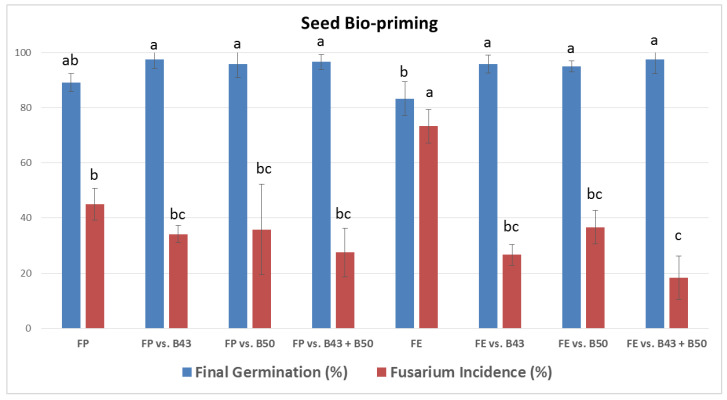
Final germination and pathogen incidence on garden pea seeds after bio-priming with selected *Bacillus* isolates (*B. subtilis* B43, *B. amyloliquefaciens* B50, and B43 + B50 consortium) and infection with *F. proliferatum* PS1 (FP) and *F. equiseti* PS18 (FE). Means (*n* = 4) with standard deviation are shown. The values sharing the same lowercase letter within the same color are not significantly different (*p* ≤ 0.05, Tukey’s HSD test).

**Figure 5:**
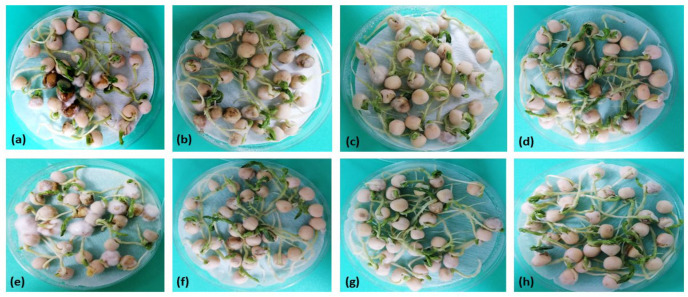
Antifungal effect of selected *Bacillus* isolates after seed bio-priming. (**a**) *F. proliferatum* PS1- treated seeds, (**b**) *B. subtilis* B43 vs. PS1-treated seeds, (**c**) *B. amyloliquefaciens* B50 vs. PS1-treated seeds, (**d**) B43 + B50 vs. PS1-treated seeds, (**e**) *F. equiseti* PS18-treated seeds, (**f**) *B. subtilis* B43 vs. PS18-treated seeds, (**g**) *B. amyloliquefaciens* B50 vs. PS18-treated seeds, (**h**) B43 + B50 vs. PS18-treated seeds.

The percentages of the final germination and pathogen incidence on seeds treated with both antagonist(s) and pathogen are presented in Figure 2, Figure 3, Figure 4 and Figure 5. All *Bacillus* treatments increased the percentage of final germination and reduced the percentage of pathogen incidence on infected seeds as compared to the positive controls (only *F. proliferatum* PS1 or *F. equiseti* PS18 treated), both after inoculation (Figure 2 and Figure 3) and bio-priming (Figure 4 and Figure 5). After seed inoculation, a significant increase in final germination was achieved with *B. subtilis* B43 and consortium (B43 + B50) as compared to PS18-treated seeds (Figure 2). The consortium also exhibited a significant impact on PS1 and PS18 incidence on garden pea seeds during both application techniques (Figure 2 and Figure 4). Moreover, examined *Bacillus* treatments significantly increased final germination and reduced the incidence of PS18 on garden pea seeds as a result of bio-priming (Figure 4).

Generally, the highest increase in final germination of *Fusarium*-treated pea seeds was recorded in consortium treatment (PS18, both application techniques), followed by *B. subtilis* B43 (PS1, seed bio-priming) and *B. amyloliquefaciens* B50 (PS1, seed inoculation) (Figure 2 and Figure 4). *B. subtilis* B43 treatment led to an increase in the final germination of PS1-treated and PS18-treated seeds after inoculation (11.7% and 19.2%) and bio-priming (8.33% and 12.5%). Additionally, *B. amyloliquefaciens* B50 increased the final germination of PS1 and PS18-treated seeds (15% for both fungi) after seed inoculation as well as after seed bio-priming (6.66% and 11.7%). The improvements in final germination by using the *Bacillus* consortium were as follows: 13.3% and 32% for PS1/PS18-treated seeds during inoculation, as well as 7.5% and 14.2% for PS1/PS18-treated seeds during bio-priming.

The incidence of *F. proliferatum* PS1 on pea seeds in the PS1 treatments (positive control) was high (seed inoculation) and moderate (seed bio-priming), whereas the incidence of *F. equiseti* PS18 was high in both PS18 controls (seed inoculation and seed bio-priming) (Figure 2 and Figure 4). Overall, the highest decrease in the incidence of both *Fusarium* strains on pea seeds was recorded in consortium treatment, followed by *B. subtilis* B43 treatment at seed bio-priming and *B. amyloliquefaciens* B50 at seed inoculation. After both seed inoculation and bio-priming, consortium (B43 + B50) treatment reduced the incidence of PS1 (15.8% and 15.5%) and PS18 (33.3% and 55.0%). A single application of *B. subtilis* B43 reduced the incidence of PS1 and PS18 during seed inoculation (9.17% and 4.16%) and seed bio-priming (10.8% and 46.7%). Furthermore, *B. amyloliquefaciens* B50 led to a decrease in PS1 and PS18 on pea seeds of 13.3% and 6.66% in the case of the inoculation method, as well as 9.17% and 36.5% in the case of bio-priming. A significant reduction in pathogen incidence was obtained only in B50 vs. PS18 treatment during seed bio-priming.

## 4. Discussion

*Bacillus* species produce various compounds involved in the biocontrol of plant diseases and the stimulation of plant growth, which makes them suitable candidates for seed protection from pathogens, along with improvements in seed germination. *Bacillus* spp. are also preferable candidates for commercialization due to the production of numerous bioactive metabolites, extremely tolerant endospores, and a higher growth rate in short periods [13]. These bacteria are commonly found in different ecological niches, including soil and plant rhizosphere. In the present study, *Bacillus* isolates were isolated from the rhizosphere of different field and vegetable crops. The bacteria were rod-shaped, catalase- and Gram-positive. Rhizosphere bacteria with multiple beneficial traits, widely known as plant growth-promoting rhizobacteria (PGPR), play important roles in growth promotion, plant nutrition, defense against pathogens, and stress alleviation [11,14]. Bacilli are among the most commonly reported PGPR, which have a wide application as biocontrol agents as well as biofertilizers [37]. 

The presence of fungi on the seeds is an important determinant of seed quality. *Fusarium* spp. are among the most resistant pathogens that infect numerous agricultural crops and cause symptoms such as stunted growth, wilting, yellowing, decay, canker, and plant death, resulting in serious yield losses (up to 80%) [38]. Seed-borne *Fusarium* spp. are of particular importance due to their impact on the overall seed health, seed germination, and crop stand in the field. In recent studies, based on the *tef-1α* sequencing, the occurrence of *F. proliferatum* and *F. equiseti* strains was confirmed on the garden pea seeds [9], as well as the presence of *F. proliferatum* and *F. graminearum* on the common bean and soybean seeds, respectively [23,24]. In general, *Fusarium* spp. are difficult to suppress since they easily prevail over host resistance to different means of control [39]. It has been shown that *Fusarium* spp. were more resistant to the biocontrol action of *Bacillus* spp. as compared to other fungal species [40]. Similarly, Miljaković et al. [32] studied the effects of *Bacillus* spp. on different fungal pathogens and found the least antagonistic effect toward *Fusarium* spp. strains. 

The most active *Bacillus* isolates were further identified based on 16S rDNA sequencing. Molecular identification proved that six selected isolates from a total of 46 belonged to the *B. subtilis* complex, with the following species: *B. velezensis* (B42), *B. subtilis* (B43), *B. mojavensis* (B44, B46), *B. amyloliquefaciens* (B50), and *B. halotolerans* (B66). Our results support the findings that the antagonistic properties mostly refer to the *B. subtilis* species complex. Moreover, species of this group, such as *B. subtilis* and *B. amyloliquefaciens*, are the most common components of commercially available biofungicides. For instance, *B. subtilis* and *B. amyloliquefaciens* devote 4–5% and 8.5% of their total genome capacity to the biosynthesis of secondary metabolites with antimicrobial potential [13]. Thus, *Bacillus* species are renowned for their ability to control plant diseases through the synthesis of hydrolytic enzymes. The main components of *Fusarium* cell walls are chitin and glucan, suggesting the responsibility of *Bacillus* chitinases and glucanases for distortions, thinning, swelling, and other hyphal abnormalities [41]. Additionally, fungal cell walls are composed of cellulose, lipids, and proteins. Thus, cellulases, lipases, and proteases produced by *Bacillus* spp. may contribute to the lysis of pathogen cell walls. In our study, all the selected bacterial isolates were tested and found to be positive for cellulase activity in vitro. Moreover, three bacterial isolates (*B. velezensis* B42, *B. subtilis* B43, and *B. amyloliquefaciens* B50) were positive for the endoglucanase gene, indicating the potential involvement of this enzyme in their antifungal activity against target fungi. However, PCR analysis did not prove the presence of the genes for chitinase production in the tested *Bacillus* isolates. Similarly, *B. subtilis*, *B. pumilus*, and *B. safensis* isolates as superior cellulase producers showed the highest antifungal activity against *F. proliferatum*, *F. tricinctum*, *F. acuminatum*, *F. oxysporum* f. sp. *cepae*, and *F. verticillioides* [31]. Furthermore, the selected *Bacillus* isolates differed from each other in the type of lipopeptide genes detected by PCR amplification. For instance, isolate *B. velezensis* B42 possessed genes for bacillomycin and iturin production; isolates of *B. mojavensis* B44 and B46 were positive for the surfactin and fengycin lipopeptide genes and the most effective isolates, *B. subtilis* B43 and *B. amyloliquefaciens* B50 had genes for surfactin and bacillomycin biosynthesis, respectively. Lipopeptide antibiotics have been linked with the inhibition of fungal pathogens through the disruption of cell membrane structure and permeability, but they have also been associated with their effect on intracellular targets [42]. Surfactin and other lipopeptides are also well known for their role in biofilm formation and cell motility, thus contributing to effective rhizosphere colonization [43]. Similarly, Blacutt et al. [44] reported the activity of surfactin and fengycin in *B. mojavensis*, which exhibited antifungal activity against *F. verticillioides* affecting maize. Additionally, Cao et al. [45] identified three lipopeptides, namely surfactin, iturin, and fengycin, as responsible for the antifungal activity of two rhizosphere-associated *B. velezensis* strains against *Fusarium oxysporum*. Moreover, Hanif et al. [46] reported that fengycin-producing *B. amyloliquefaciens* can also inhibit *F. graminearum* and its mycotoxins. Considering that genes for the examined lipopeptides were not detected in the isolate *B. halotolerans* B66, it was assumed that its antagonism is due to hydrolytic enzymes and siderophores. Similarly, *B. halotolerans* endophytes, which showed cellulase, chitinase, amylase, and siderophore production, proved active against sixteen *Fusarium* isolates belonging to *F. oxysporum*, *F. solani*, *F. acuminatum*, and *F. chlamydosporum* [47]. 

The present study demonstrated that all six *Bacillus* spp. isolates (B42, B43, B44, B46, B50, and B66) showed IAA production, although the concentration of this auxin varied depending on the precursor concentration in the medium as well as the examined isolate. As the main auxin in plants, IAA regulates different aspects of plant growth and development. Particularly, seed treatment with IAA-producing bacteria improves seed germination, initial seedling growth, and seedling vigor, resulting in higher plant resistance to various stresses during and after emergence [48]. This trait may be related to the stimulating effect of examined *Bacillus* isolates on seed germination, root and shoot length and weight, and seedling vigor index of garden peas. In fact, *Bacillus* isolates that produced the highest amount of IAA had the best effect on tested germination parameters. Furthermore, IAA-producing bacteria use this hormone in diverse interactions with plants, such as colonization, stimulation, defense responses, and as a microbial signaling and metabolic molecule [49]. In addition, the results showed that all the selected *Bacillus* isolates synthesize siderophores, which make iron unavailable to soil-borne pathogens, thus reducing their metabolic activity and growth [50]. For instance, IAA and siderophore-producing *B. subtilis* isolates exhibited antifungal activity against *Fusarium graminearum*, *F. subglutinans*, *Diaporthe caulivora*, *D. sojae*, *D. eres*, *D. longicolla*, and *Macrophomina phaseolina* and significantly improved the germination parameters of two soybean cultivars [32]. The present study also showed that *Bacillus* isolates were able to dissolve organic phosphates (P mineralization), suggesting their potential to convert insoluble phosphorus into plant-available forms. It was found that the *B. subtilis* strain, with its ability to produce IAA, siderophores, and lytic enzymes and solubilize organic and inorganic phosphates and zinc, significantly inhibited the growth of different fungi, including *Fusarium oxysporum*, and improved the seedling growth of maize and rice [51]. The same study indicated the expression of 114 genes in *B. subtilis*, among which 10%, 32%, and 10% were involved in antibiosis, metabolism, and nutrient transportation, respectively [51]. 

Seed germination and seedling emergence are pivotal for reaching optimal crop establishment and high yields. In the initial stages of development, plants are very sensitive to the undesirable effects of biotic factors, such as infection of seeds or seedlings with soil- and seed-borne pathogens. Seed treatment with bacterial inoculum as a potential biofungicide before infection can contribute to seed protection and enhance germination in the presence of a fungal pathogen [52]. Isolates *B. subtilis* B43 and *B. amyloliquefaciens* B50 showed high antifungal activity against *Fusarium* strains in vitro (Table 1), while their effect, individually or combined, on the protection of garden pea seeds from infection during seed inoculation and seed bio-priming was also very pronounced (Figure 2 and Figure 4). All *Bacillus* treatments improved the final germination and reduced the pathogen incidence on infected seeds, both after inoculation and bio-priming. Mostly, the highest effect on the suppression of seed infection and improvement of pea germination in the presence of *Fusarium* strains was achieved by the *B. subtilis* B43 and *B. amyloliquefaciens* B50 consortium. The application of consortia-based bacterial inoculants could contribute to their competence, survival, and performance, and thereby, their influence on plant growth and responses to biotic and abiotic stresses [32]. In this study, *F. equiseti* PS18 was more pathogenic, although the effect of applied bacterial isolates after both application techniques was better and more noticeable against this strain as compared to *F. proliferatum* PS1. In fact, a significant effect of the consortium was recorded on the final germination and the pathogen incidence of PS18-treated seeds during both application methods. Also, the consortium significantly reduced the PS1 incidence after seed inoculation. Additionally, a single application of B43 and B50 led had a significant effect on both examined parameters of PS18-treated seeds during bio-priming. Bio-priming treatments promote rapid and uniform seed germination and seedling emergence under adverse conditions, such as the presence of plant pathogens. This is in agreement with the findings of this research since bio-priming generally gave higher final germination values as compared to seed inoculation. Similarly, Miljaković et al. [35] reported that bio-priming of soybean seeds with *B. japonicum* and *B. megaterium* improved seed germination and initial plant growth. In this study, *Bacillus* isolates had a better effect, i.e., led to a greater reduction in the pathogen(s) incidence on the seeds in the case of bio-priming. Consistently, Naik [53] demonstrated that bio-priming with *Trichoderma viride* or *Pseudomonas fluorescens* efficiently suppressed seed-borne pathogens (*Fusarium* sp., *Aspergillus niger*, *A. flavus*, and *Penicillium* sp.) and improved seed quality in the garden pea. Bio-priming enhances bacterial adhesion and encapsulation on the seeds while triggering seed enzymes, hormones, and overall metabolic activities [54]. It mitigates the adverse effects of stress by improving the physiological functioning of seeds through the activation of reactive oxygen species (ROS) and the accumulation of antioxidative enzymes [15]. Seed bio-priming also enables DNA and protein synthesis and assists in mitochondrial development [15]. Thus, bio-priming improves seed and overall plant health through improved nutrient uptake and host resistance to both stresses, contributing to higher seed germination, seedling vigor, and emergence [15]. This application technique has shown beneficial effects on germination, growth, and yield in numerous crops (soybean, wheat, barley, maize, sunflower, tomato, etc.) and against a wide variety of pathogens (*Fusarium*, *Colletotrichum*, *Verticillium*, *Rhizoctonia*, *Sclerotium*, etc.) [55]. The application of beneficial bacteria as priming agents has been reported to promote stimulus in the rhizosphere, facilitate nutrient cycling, accumulate enzymatic activity, and thereby improve crop performance under adverse environmental conditions [56]. The antagonism and positive effects on germination, both in the absence and presence of pathogens, are probably related to the production of lipopeptides, namely surfactin (*B. subtilis* B43) and bacillomicin (*B. amyloliquefaciens* B50), cellulase, IAA, and siderophores. Similarly, the significant increase in shoot and root length and fresh weight, along with the reduction in root rot caused by *Fusarium solani* was recorded in pea plants inoculated with *B. subtilis* and *B. halotolerans*, which produced the lipopeptides surfactin and fengycin, the hydrolytic enzymes protease and glucanase, and siderophores [57]. *B. subtilis* exhibited the highest antifungal activity (40% inhibition in fungal growth) against *Fusarium oxysporum* f. sp. *conglutinans*, *F. oxysporum* f. sp. *matthioli*, and *F. solani* when confronted in vitro, as compared to other *Bacillus* species [41]. Additionally, the inoculation of pea seeds with the abovementioned strain led to a reduction in wilt severity in plants, along with a 35% increase in dry plant biomass in relation to non-treated plants cultivated in soil infested with *Fusarium*. To our knowledge, this is the first research on the use of indigenous *Bacillus* rhizosphere isolates for suppression of *F. proliferatum* and *F. equiseti* strains originating from infected garden pea seeds. Newly isolated *Bacillus* spp. from the rhizosphere of different field and vegetable crops generally had good biocontrol and plant growth-promoting potential. *B. subtilis* B43 and *B. amyloliquefaciens* B50 had the highest effect on seed germination of garden peas, both in the absence and presence of the *Fusarium* pathogenic strains. Best-performing isolates could be prominent candidates for *Bacillus*-based formulations intended for the production of chemical-free peas and other leguminous vegetable crops. Notwithstanding the encouraging results, additional field experiments will be required to determine which strains are the most effective in different environments, both as single and combined inoculants. Further studies should also consider all the limitations and challenges related to the lab-to-field transition, suitable formulations, and application methods to ensure the desired effectiveness of bio-based fungicides in natural conditions.

## Figures and Tables

**Table 1:** Antifungal activity of *Bacillus* spp. isolates against *Fusarium* spp. strains.

Isolate	Fungal Growth Inhibition (%)					Isolate	Fungal Growth Inhibition (%)				
	PS1	P1	PS18	S1	Average		PS1	P1	PS18	S1	Average
B33	13.3 ^cde^	23.3 ^n^	–	–	9.15	B56	–	–	–	–	–
B34	12.2 ^de^	28.9 ^k–n^	7.78 ^hi^	–	12.2	B57	13.3 ^cde^	28.9 ^k–n^	7.78 ^hi^	24.4 ^d–j^	18.6
B35	30.0 ^a–e^	36.7 ^e–m^	22.2 ^b–i^	30.0 ^c–i^	29.7	B58	34.4 ^a–e^	45.6 ^a–g^	25.6 ^a–h^	–	26.4
B36	20.0 ^cde^	30.0 ^j–n^	10.0 ^ghi^	28.9 ^c–j^	22.2	B59	–	46.7 ^a–g^	–	33.3 ^a–g^	20.0
B37	27.8 ^a–e^	27.8 ^l–n^	14.4 ^f–i^	28.9 ^c–j^	24.7	B60	23.3 ^a–e^	35.6 ^f–n^	15.6 ^f–i^	33.3 ^a–g^	27.0
B38	28.9 ^a–e^	34.4 ^g–n^	22.2 ^b–i^	31.1 ^c–h^	29.2	B61	–	48.9 ^a–e^	–	24.4 ^d–j^	18.3
B39	21.1 ^b–e^	24.4 ^mn^	11.1 ^f–i^	23.3 ^e–j^	19.9	B62	10.0 ^de^	28.9 ^k–n^	3.33 ^i^	31.1 ^c–h^	18.3
B40	25.6 ^a–e^	35.6 ^f–n^	6.67 ^hi^	26.7 ^d–j^	23.6	B63	17.8 ^cde^	32.2 ^h–n^	10.0 ^ghi^	31.1 ^c–h^	22.8
B41	30.0 ^a–e^	35.6 ^f–n^	24.4 ^b–h^	13.3 ^jk^	25.8	B64	26.7 ^a–e^	47.8 ^a–f^	16.7 ^e–i^	14.4 ^ijk^	26.4
B42	42.2 ^abc^	37.8 ^d–l^	41.1 ^ab^	37.8 ^a–e^	**39.7**	B65	7.78 ^e^	46.7 ^a–g^	2.22 ^i^	36.7 ^a–f^	23.3
B43	42.2 ^abc^	43.3 ^a–h^	38.9 ^abc^	47.8 ^ab^	**43.1**	B66	52.2 ^a^	40.0 ^c–l^	37.8 ^a–d^	48.9 ^a^	**44.7**
B44	51.1 ^a^	47.8 ^a–f^	36.7 ^a–e^	40.0 ^a–d^	**43.9**	B67	–	36.7 ^e–m^	–	–	9.17
B45	–	24.4 ^mn^	–	18.9 ^g–k^	10.8	B68	21.1 ^b–e^	53.3 ^ab^	12.2 ^f–i^	36.7 ^a–f^	30.8
B46	35.6 ^a–e^	55.6 ^a^	31.1 ^a–f^	40.0 ^a–d^	**40.6**	B69	7.78 ^e^	28.9 ^k–n^	–	36.7 ^a–f^	18.3
B47	–	31.1 ^i–n^	–	38.9 ^a–e^	17.5	B70	24.4 ^a–e^	52.2 ^abc^	16.7 ^e–i^	–	23.3
B48	27.8 ^a–e^	38.9 ^d–l^	21.1 ^b–i^	36.7 ^a–f^	31.1	B71	21.1 ^b–e^	44.4 ^a–h^	13.3 ^f–i^	27.8 ^c–j^	26.6
B49	12.2 ^de^	36.7 ^e–m^	–	25.6 ^d–j^	18.6	B72	33.3 ^a–e^	52.2 ^abc^	21.1 ^b–i^	21.1 ^f–j^	31.9
B50	50.0 ^ab^	45.6 ^a–g^	45.6 ^a^	40.0 ^a–d^	**45.3**	B73	17.8 ^cde^	31.1 ^i–n^	13.3 f–i	–	15.5
B51	28.9 ^a–e^	48.9 ^a–e^	14.4 ^f–i^	3.33 ^k^	23.8	B74	30.0 ^a–e^	47.8 ^a–f^	20.0 ^c–i^	28.9 ^c–j^	31.7
B52	–	–	–	–	–	B75	37.8 ^a–d^	41.1 ^b–k^	5.56 ^hi^	43.3 ^abc^	31.9
B53	14.4 ^cde^	23.3 ^n^	14.4 ^f–i^	16.7 ^h–k^	17.2	B76	35.6 ^a–e^	42.2 ^b–j^	28.9 ^a–g^	33.3 ^a–g^	35.0
B54	25.6 ^a–e^	27.8 ^l–n^	21.1 ^b–i^	32.2 ^b–h^	26.7	B77	24.4 ^a–e^	53.3 ^ab^	24.4 ^b–h^	43.3 ^abc^	36.3
B55	21.1 ^b–e^	50.0 ^a–d^	17.8 ^d–i^	26.7 ^d–j^	28.9	B78	28.9 ^a–e^	42.2 ^b–j^	25.6 ^a–h^	27.8 ^c–j^	31.1

Means (*n* = 3) of percent of inhibition of fungal growth are shown. The values sharing the same lowercase letters within the column are not significantly different (*p* ≤ 0.05, Tukey’s HSD test). The values in bold represent the highest average percent of inhibition for tested *Fusarium* strains as a result of the action of a particular *Bacillus* isolate. PS1: *F. proliferatum* (garden pea); P1: *F. proliferatum* (common bean); PS18: *F. equiseti* (garden pea); S1: *F. graminearum* (soybean).

**Table 2:** Selected *Bacillus* spp. isolates and their isolation sources.

Isolate	Species	Accesion Number	Rhizosphere	Locality	GPS Coordinates
B42	*B. velezensis*	OL636363	Common bean	Đurđevo, South Bačka District, Serbia	45°19′28″ N 20°03′52″ E
B43	*B. subtilis*	OR875367	Soybean	Rimski šančevi, South Bačka District, Serbia	45°19′10″ N 19°50′22″ E
B44	*B. mojavensis*	OR875368	Maize	Orom, North Banat District, Serbia	45°58′59″ N 19°49′59″ E
B46	*B. mojavensis*	OR875369	Soybean	Lipar, West Bačka District, Serbia	45°36′31″ N 19° 21′31″ E
B50	*B. amyloliquefaciens*	OR875370	Garden pea	Rimski šančevi, South Bačka District, Serbia	45°19′10″ N 19°50′22″ E
B66	*B. halotolerans*	OR875371	Soybean	Mali Iđoš, North Bačka district, Serbia	45°42′43″ N 19°39′26″ E

**Table 3:** Antagonistic traits of selected *Bacillus* spp. isolates.

Isolate	Lytic Enzymes			Cyclic Lipopeptides *			
	Cellulase	Chitinase *	Endoglucanase *	Surfactin	Bacillomycin	Iturin	Fengycin
*B. velezensis* B42	+++	−	+	−	+	+	−
*B. subtilis* B43	+++	−	+	+	−	−	−
*B. mojavensis* B44	+++	−	−	+	−	−	+
*B. mojavensis* B46	+++	−	−	+	−	−	+
*B. amyloliquefaciens* B50	+++	−	+	−	+	−	−
*B. halotolerans* B66	++	−	−	−	−	−	−

Cellulase: (+) 1–5 mm wide of halo zone, (++) 5–10 mm wide of halo zone, (+++) 10–15 mm wide of halo zone. * Genes encoding endoglucanase, chitinase (*ChiA*), surfactin (*Sfp*), bacillomycin (*BamC*), iturin (*ItuA-ItuB*), and fengycin (*FenD*); production: (+) positive test result; (−) negative test result.

**Table 4:** PGP traits of selected *Bacillus* isolates.

Isolate	IAA (µg mL^−1^ ± SD) at 250 µg mL^−1^ L-Tryptophan	IAA (µg mL^−1^ ± SD) at 500 µg mL^−1^ L-Tryptophan	P Solubilization	P Mineralization	Siderophores
*B. velezensis* B42	2.60 ± 0.10 ^d^	7.07 ± 0.21 ^d^	–	+	+
*B. subtilis* B43	14.73 ± 0.31 ^a^	15.70 ± 0.26 ^a^	–	++	+
*B. mojavensis* B44	1.87 ± 0.06 ^d^	2.80 ± 0.75 ^e^	–	+	+
*B. mojavensis* B46	5.15 ± 0.05 ^c^	8.35 ± 0.05 ^c^	–	++	++
*B. amyloliquefaciens* B50	8.27 ± 0.75 ^b^	11.00 ± 0.36 ^b^	–	+	++
*B. halotolerans* B66	2.27 ± 0.23 ^d^	6.67 ± 0.12 ^d^	–	+	+

Means (*n* = 3) of indol-3-acetic acid (IAA) concentration are shown. The values sharing the same lowercase letter within the column are not significantly different (*p* ≤ 0.05, Tukey’s HSD test). P Solubilization/Mineralization, Siderophores: (+) 1–5 mm wide of zone, (++) 5–10 mm wide of zone; (–) not detected.

**Table 5:** Effect of selected *Bacillus* isolates on germination and the initial growth of garden pea.

Treatment	Final Germination (%)	Shoot Length (mm)	Root Length (mm)	Shoot Dry Weight (g)	Root Dry Weight (g)	Seedling Vigor Index
Control	90.67 ^abc^	39.50 ^b^	103.50 ^c^	0.174 ^c^	0.182 ^b^	1296.43 ^c^
*B. velezensis* B42	92.00 ^abc^	40.17 ^b^	102.83 ^c^	0.182 ^bc^	0.188 ^ab^	1315.70 ^c^
*B. subtilis* B43	95.00 ^a^	43.00 ^b^	**127.83** ^a^	**0.197** ^ab^	0.200 ^ab^	**1622.08** ^a^
*B. mojavensis* B44	92.67 ^ab^	35.67 ^c^	104.00 ^c^	0.178 ^bc^	0.198 ^ab^	1294.65 ^c^
*B. mojavensis* B46	89.00 ^bc^	42.33 ^b^	**118.17** ^b^	0.189 ^abc^	0.199 ^ab^	**1428.57** ^b^
*B. amyloliquefaciens* B50	93.33 ^ab^	**51.50** ^a^	**118.67** ^b^	**0.208** ^a^	**0.210** ^a^	**1588.00** ^a^
*B. halotolerans* B66	87.33 ^c^	40.33 ^b^	**117.33** ^b^	0.189 ^abc^	0.197 ^ab^	1376.80 ^bc^
*p*	0.0012	0.0000	0.0000	0.0029	0.0451	0.0000

Means of (*n* = 3) germination parameters are shown. The values sharing the same lowercase letter within the column are not significantly different (*p* ≤ 0.05, Tukey’s HSD test). The values in bold represent a significant increase over control as a result of the action of a particular *Bacillus* isolate.

## Data Availability

Data are contained within the article and Supplementary Materials.

## Supplementary Materials

The following supporting information can be downloaded at: https://www.mdpi.com/article/10.3390/jof10050358/s1, Figure S1: Polymerase chain reaction (PCR) screening for 16S rDNA gene (~1460 bp) in selected *Bacillus* spp. isolates (B42, B43, B44, B46, B50, B66); Figure S2: Phylogenetic tree based on the neighbor-joining (NJ) analysis of 16S rDNA gene sequences for selected *Bacillus* spp. isolates (*B. velezensis* B42; *B. subtilis* B43; *B. mojavensis* B44, B46; *B. amyloliquefaciens* B50; *B. halotolerans* B66) and related *Bacillus* spp. strains from the NCBI database; Table S1: Primers and corresponding genes for PCR detection of lytic enzymes and cyclic lipopeptides; Figure S3: Polymerase chain reaction (PCR) screening for endoglucanase biosynthetic gene (~1311 bp) in *Bacillus* spp. isolates (B35–B45); Figure S4: Polymerase chain reaction (PCR) screening for endoglucanase biosynthetic gene (~1311 bp) in *Bacillus* spp. isolates (B46–B56); Figure S5: Polymerase chain reaction (PCR) screening for surfactin biosynthetic gene (~675 bp) in *Bacillus* spp. isolates (B43–B52); Figure S6: Polymerase chain reaction (PCR) screening for bacillomycin biosynthetic gene (~875 bp) in *Bacillus* spp. isolates (B35–B45); Figure S7: Polymerase chain reaction (PCR) screening for bacillomycin biosynthetic gene (~875 bp) in *Bacillus* spp. isolates (B46–B56); Figure S8: Polymerase chain reaction (PCR) screening for iturin biosynthetic gene (~2000 bp) in *Bacillus* spp. isolates (B35–B45); Figure S9: Polymerase chain reaction (PCR) screening for fengycin biosynthetic gene (~964 bp) in *Bacillus* spp. isolates (B35–B45); Figure S10: Polymerase chain reaction (PCR) screening for fengycin biosynthetic gene (~964 bp) in *Bacillus* spp. isolates (B46–B56).
