# Supplementary material for: Bio-Priming with Bacillus Isolates Suppresses Seed Infection and Improves the Germination of Garden Peas in the Presence of Fusarium Strains
Source: J Fungi (Basel). 2024 May 17;10(5):358. doi: 10.3390/jof10050358 (PMC11122518; doi:10.3390/jof10050358)
Supplement: Supplementary file 1 [file jof-10-00358-s001.zip › jof-2975788-supplementary.pdf]

Supplementary Material

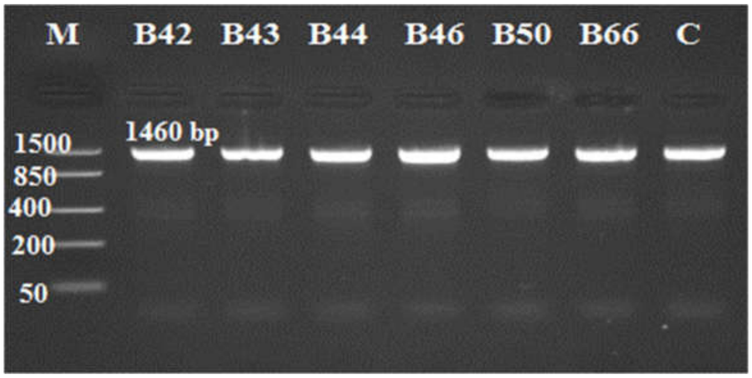

Supplementary Materials Figure S1. Polymerase chain reaction (PCR) screening for 16S rDNAgene (~1460 bp) in selected *Bacillus* spp. isolates (B42, B43, B44, B46, B50, B66). (M) PCR marker (Step Ladder, 50–1500 bp), (C) positive control (*B. subtilis* B32).

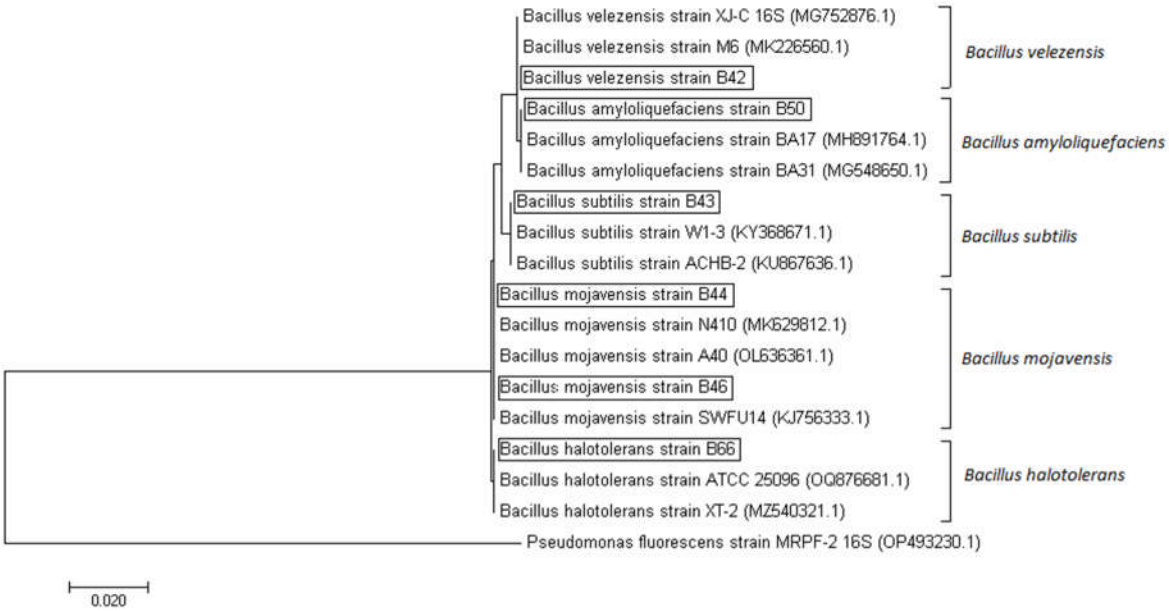

Supplementary Materials Figure S2. Phylogenetic tree based on the neighbor-joining (NJ) analysis of 16S rDNA gene sequences for selected *Bacillus* spp. isolates (*B. velezensis* B42; *B. subtilis* B43; *B. mojavensis* B44, B46; *B. amyloliquefaciens* B50; *B. halotolerans* B66) and related *Bacillus* spp. strains from the NCBI database.

Supplementary Materials Table S1. Primers and corresponding genes for PCR detection of lytic enzymes and cyclic lipopeptides

Target Gene	Primers	Sequence (5′-3)	Amplicon size (bp)	Reference
ChiA	Qchi-f	5′-GATATCGACTGGGAGTTCCC-3′	~225	[29]
	Qchi-r	5′-CATAGAAGTCGTAGGTCATC-3′		
Endoglucanase	EN1F	5′-CCAGTAGCCAAGAATGGCCAGC-3′	~1311	[30]
	EN1R	5′-GGAATAATCGCCGCTTTGTGC-3′		
Sfp	Sfp-f	5′-ATGAAGATTTACGGAATTTA-3′	~675	[29]
	Sfp-r	5′-TTATAAAAGCTCTTCGTACG-3′		
BamC	Bacc1-f	5′ -GAAGGACACGGAGAGAGTC-3′	~875	[29]
	Bacc1-r	5′-CGTGATGACTGTTCATGCT-3′		
ItuA-ItuB	ITUP1-F	5'-AGCTTAGGGAACAATTGTCATCGGGGCTTC-3'	~2000	[27]
	ITUP1-R	5'-TCAGATAGGCCGCCATATCGGAATGATTCG-3'		
FenD	FenD1-f	5′-TTTGGCAGCAGGAGAAGTTT-3′	~964	[29]
	FenD1-r	5′-GCTGTCCGTTCTGCTTTTTC-3′		

ChiA, endoglucanase, *Sfp*, *BamC*, *ItuA-ItuB*, and *FenD*: Genes encoding for chitinase, endoglucanase, surfactin, bacillomycin D, parts of the genes *ituA* and *ituB* and intergenic sequences between them, and fengycin , respectively.

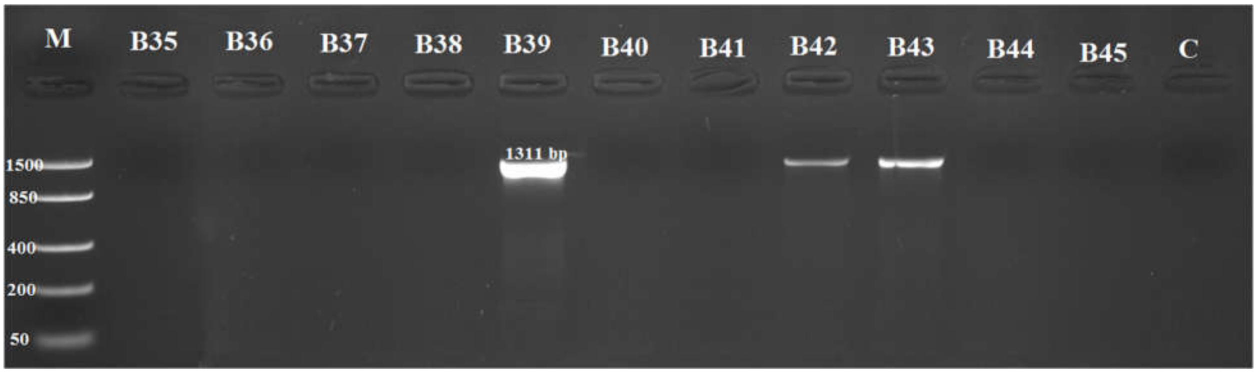

Supplementary Materials Figure S3. Polymerase chain reaction (PCR) screening for endoglucanase biosynthetic gene (~1311 bp) in *Bacillus* spp. isolates (B35–B45). (M) PCR marker (Step Ladder, 50–1500 bp), (C) negative control.

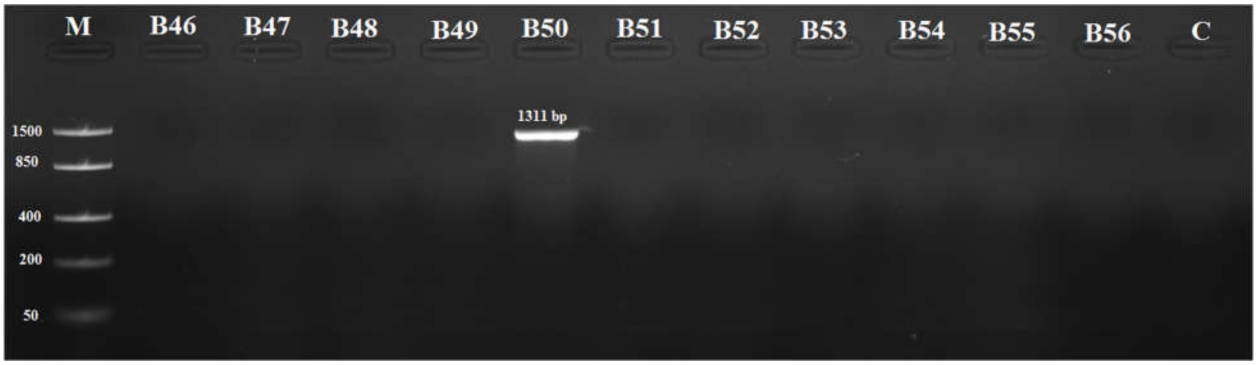

Supplementary Materials Figure S4. Polymerase chain reaction (PCR) screening for endoglucanase biosynthetic gene (~1311 bp) in *Bacillus* spp. isolates (B46–B56). (M) PCR marker (Step Ladder, 50–1500 bp), (C) negative control.

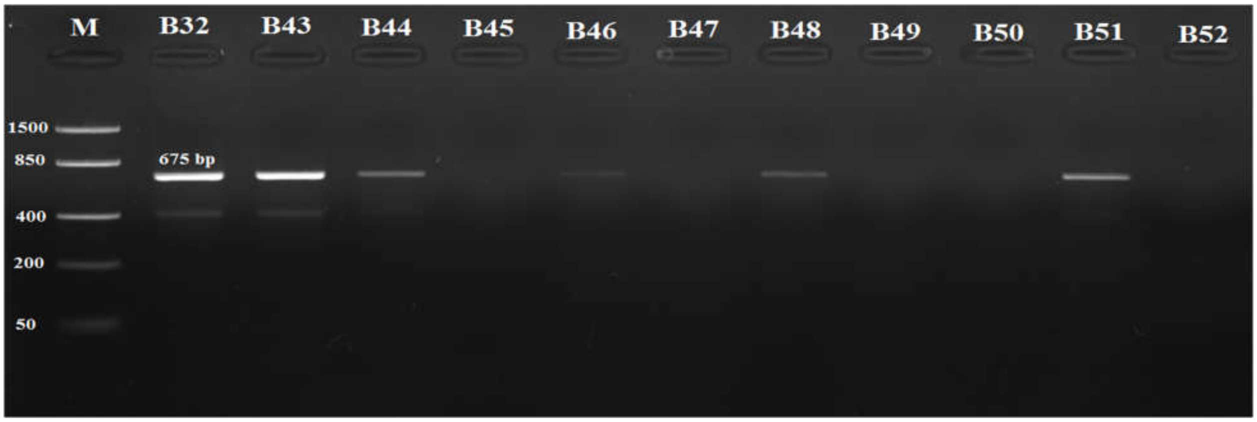

Supplementary Materials Figure S5. Polymerase chain reaction (PCR) screening for surfactin biosynthetic gene (~675 bp) in *Bacillus* spp. isolates (B43–B52). (M) PCR marker (Step Ladder, 50–1500 bp), (B32) positive control, (C) negative control.

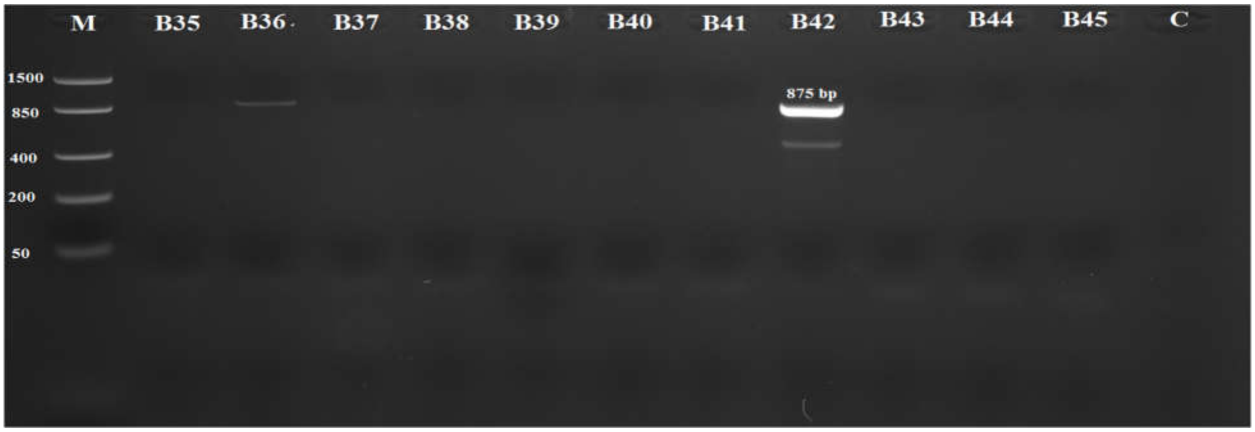

Supplementary Materials Figure S6. Polymerase chain reaction (PCR) screening for bacillomycin biosynthetic gene (~875 bp) in *Bacillus* spp. isolates (B35–B45). (M) PCR marker (Step Ladder, 50–1500 bp), (C) negative control.

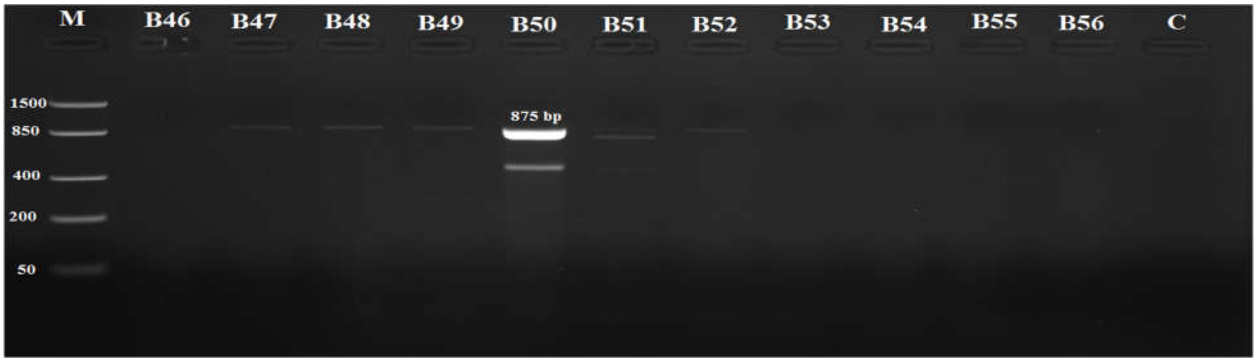

Supplementary Materials Figure S7. Polymerase chain reaction (PCR) screening for bacillomycin biosynthetic gene (~875 bp) in *Bacillus* spp. isolates (B46–B56). (M) PCR marker (Step Ladder, 50–1500 bp), (C) negative control.

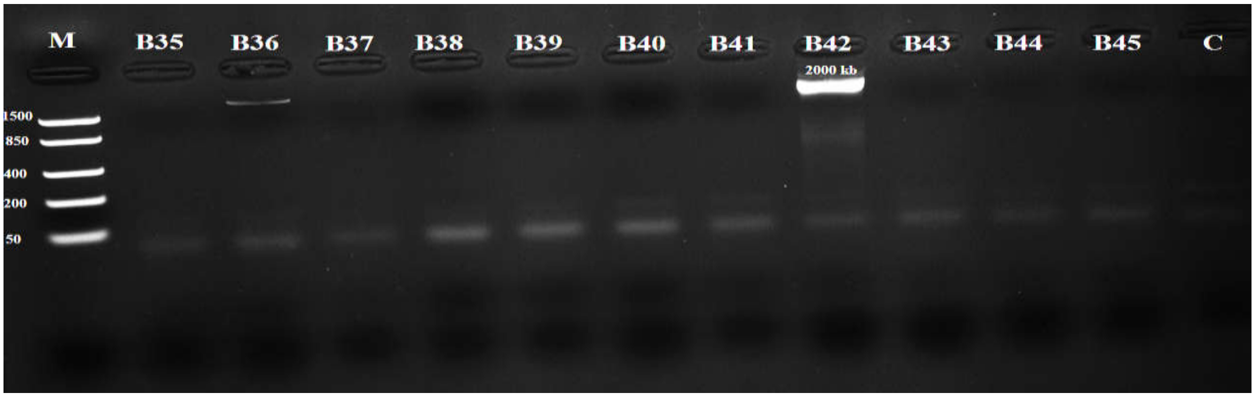

Supplementary Materials Figure S8. Polymerase chain reaction (PCR) screening for iturin biosynthetic gene (~2000 bp) in *Bacillus* spp. isolates (B35–B45). (M) PCR marker (Step Ladder, 50–1500 bp), (C) negative control.

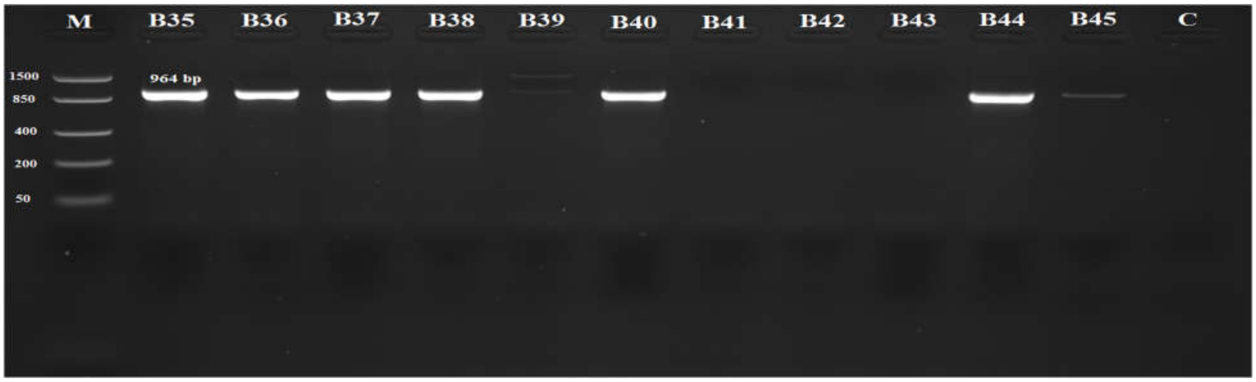

Supplementary Materials Figure S9. Polymerase chain reaction (PCR) screening for fengycin biosynthetic gene (~964 bp) in *Bacillus* spp. isolates (B35–B45). (M) PCR marker (Step Ladder, 50–1500 bp), (C) negative control.

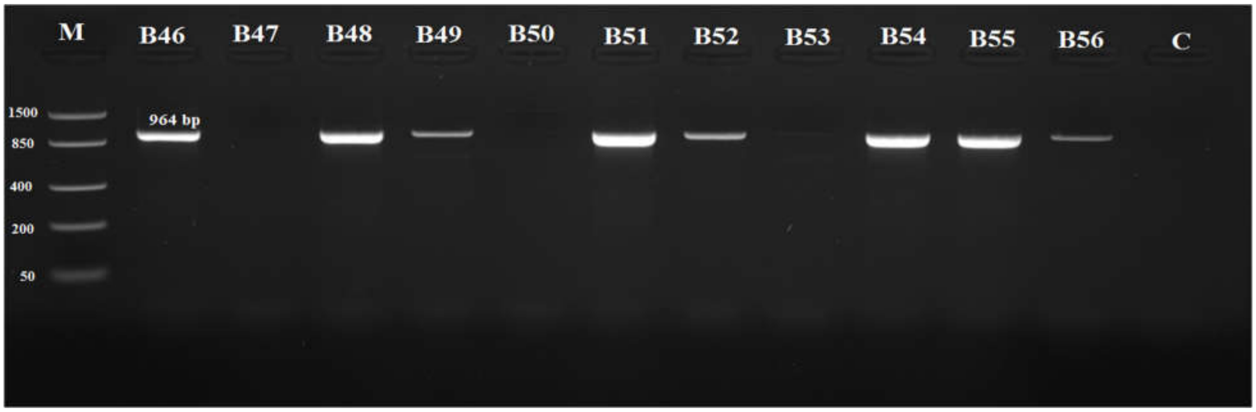

Supplementary Materials Figure S10. Polymerase chain reaction (PCR) screening for fengycin biosynthetic gene (~964 bp) in *Bacillus* spp. isolates (B46–B56). (M) PCR marker (Step Ladder, 50–1500 bp), (C) negative control.
